# The frequency distribution of vitamin D Receptor *fok* I gene polymorphism among Ugandan pulmonary TB patients

**DOI:** 10.12688/f1000research.9109.1

**Published:** 2016-07-29

**Authors:** Ester L. Acen, William Worodria, Peter Mulamba, Andrew Kambugu, Joseph Erume

**Affiliations:** 1 Department of Physiology, Makerere University College of Health Sciences, Kampala, P.O. Box 7072, Uganda; 2 Pulmonary Unit, Mulago National Referral and Teaching Hospital, Kampala, P.O.Box 7051, Uganda; 3 Department of Medicine, Makerere University College of Health Sciences, Kampala, P.O. Box 7072, Uganda; 4 Department of Agricultural and Biosystems Engineering, Makerere University College of Agricultural and Environmental Science, Kampala, P.O. Box 7062, Uganda; 5 Infectious Diseases Institute, College of Health Sciences Makerere University, Kampala, P.O Box 22418, Uganda; 6 Animal Resources and Bio-Security, Department of Bimolecular Resources and Biolab Sciences, Makerere University College of Veterinary Medicine, Kampala, P.O Box 7062, Uganda

**Keywords:** Vitamin D Receptor, Polymorphism, Fok I genotypes, Tuberculosis, Uganda Introduction

## Abstract

**Background:** *Mycobacterium tuberculosis* (TB) is still a major problem globally and especially in Africa. Vitamin D deficiency has been linked to TB in the past and studies have found vitamin D deficiency to be common among Ugandan TB patients. The functional activity of vitamin D is dependent on the genotype of the vitamin D receptor (VDR) polymorphic genes. Recent findings have indicated that VDR polymorphisms may cause increased resistance or susceptibility to TB. The vitamin D ligand and its receptor play a pivotal role in innate immunity by eliciting antimicrobial activity, which is important in prevention of TB. The *fok I* vitamin D receptor gene has extensively been examined in TB patients but findings so far have been inconclusive.

**Objectives**: This study sought to investigate the frequency distribution of the VDR *fok I *gene polymorphisms in pulmonary TB patients and controls.

**Methods**: A pilot case control study of 41 newly diagnosed TB patients and 41 healthy workers was set up. Vitamin D receptor *fok* I gene was genotyped.

**Results: **The frequency distribution of *fok I *genotype in Ugandan TB patients was 87.8% homozygous-dominant (FF), 7.3% (Ff) heterozygous and 4.8% (ff) homozygous recessive. For normal healthy subjects the frequencies were (FF) 92.6%, (Ff) 2.4% and (ff) 4.8%. No significant difference was observed in the FF and ff genotypes among TB patients and controls. The Ff heterozygous genotype distribution appeared more in TB patients than in controls. A significant difference was observed in the *fok I* genotype among gender p value 0.02. No significant difference was observed in ethnicity, p value 0.30.

**Conclusions:** The heterozygous Ff *fok I* genotype may be associated with TB in the Ugandan population.

## Introduction

According to the World Health Organization (WHO) report on the ‘Use of high burden country lists for TB by WHO in the post-2015 era (WHO/HTM/TB/2015.29), Uganda was removed from the 22 high TB burden countries. However Uganda is still among the 41 high TB/human immunodeficiency virus (HIV) burden countries ^1^ and TB remains a major public health problem. Although it is still unclear how individuals develop active TB ^2^ there is emerging evidence of multi drug resistant TB (MDRTB). Genetic predisposition to TB has been suggested in several studies ^3, 4^ but the immunological associations with genetic polymorphisms are still unclear ^5^. Vitamin D insufficiency is common worldwide and recent studies have shown that vitamin D insufficiency is associated with a higher risk of active TB. The vitamin D receptor gene (VDR) has been identified as a candidate gene for TB susceptibility, however; studies reporting from different ethnic groups have been inconsistent ^6^. Various VDR polymorphisms have been identified and associated with TB susceptibility or resistance ^2, 7, 8^. Similarly the widely studied and functional single nucleotide polymorphism (SNP) rs2228570 of VDR *fok* I gene has shown variations. This polymorphism is due to the alteration in one of the start codons in which the amino acid thymine is replaced with cytosine (T/C). The dominant homozygous FF variant has high transcriptional activity with three amino acids less since translation starts at the second codon, while the homozygous recessive (ff) has 427 amino acids and is the longer form. The absence or presence of the restriction site is designated as F or f respectively ^7, 11^. This study investigated the frequency distribution of VDR *fok I* gene among TB patients and compared it with controls in a Ugandan population.

## Methods

After obtaining permission and informed consent (see ethical considerations) a pilot study in newly diagnosed smear positive TB patients and healthy controls was conducted between the months of April and June 2013 at the Mulago National Referral and Teaching Hospital located in North of Kampala, Uganda. By consecutive sampling adult patients who presented with persistent cough for more than 3 weeks had a positive Ziehl-Nielsen smear test for the first time and signed a written consent were considered eligible. Health workers and medical trainees who worked at the TB out patient’s clinic and other wards exposed to TB were matched for sex and age with the control group. All subjects were screened for human immunodeficiency virus (HIV) and data and consent forms were filled. Blood was then collected into tubes with ethylenediaminetetraacetic acid (EDTA) anticoagulant (Becton, Dickinson and company New Jersey USA) and whole blood samples were stored at 2–8°C before DNA column extraction.

### Genotyping of *fok I* gene

Genotyping of VDR *fok I* gene was performed by PCR–direct sequencing method on ABI 310 sequencer. Human genomic DNA was extracted using the Genotype DNA isolation kit version 2.0 (HAIN Life Science, Germany) according to the manufacturer’s instructions. To check the purity genomic DNA was run on a 1.0% agarose gel in a 1 × Tris Acetate EDTA (TAE) buffer containing ethidium bromide for 1 hour at 120 volts and visualized under an UV transilluminator. The primer sequences used in our study were forward A 5’-GC TGG CCC TGG CAC TGA CTC TG TCT -3’ and reverse 5’-ATG GAA ACA CCT TGC TTC TTC TCC CTC as -3’ described by Harris *et al.* (1997). PCR was performed in 15.1 µl reaction volumes which contained 7 µl of PCR water, 1 µl of 25 mM Mgcl _2_, 1 µl of 10X master mix (Fisher biotec company Australia), 1 µl of 0.22 mg of each of the forward and reverse primers (Integrated DNA technologies company USA), 0.1 µl of 5U of Pre heated Taq polymerase and finally 5 µl of pure DNA were added and the reaction was thoroughly mixed. The PCR GTQ Thermocycler programme involved an initial denaturation at 95°C for 5 minutes, followed by 30 cycles 70°C annealing for 45 seconds, elongation at 72°C for 1 minute and a final elongation at 72°C, for 10 minutes. Amplified DNA was purified using an extraction kit (JET quick company, USA) according to manufacturer’s instructions. Cycle sequencing PCR was performed using the Big Dye terminator KIT version 3, in the Thermocycler Gene Amp PCR system 9700. The cycling programme involved denaturation at 96°C for 1 minute for 1 cycle ,annealing at 96°C for 10 minutes 30 cycles, elongation at 70°C for 30 seconds and a final elongation at 60°C for 5 minutes. The Dye EX 2.0 spin kit (250) (QIAGEN, Germany) was used to remove dye and other PCR products following the manufacturer’s instructions. Sequencing of the PCR products was done using the ABI Big Dye Termination kit (Applied Biosystems, USA) and the ABI prism 310 Genetic analyzer (Applied Biosystems). Sequences obtained were compared to those in the Gene Bank database by applying BLAST; NCBI tool. Finally DNA baser sequence assembly software version 4.7.0 bioinformatics tool was used for the Identification of *fok I* gene polymorphism. Polymorphism of FF genotype was identified on sequences with a start codon of ACG instead of an ATG followed by an ATG downstream. The homozygous ff had a start codon of ATG and consequently another ATG in the sequence. The heterozygous Ff genotype had an ACG, ACG and an ATG. 

### Statistical analysis

The sample size was calculated based on data of a previous study ^9^ with mean of 78.3 for TB and 83.5 for healthy controls and a total of 82 samples at a power of 80% would detect an effect. We therefore divided the samples into cases and controls for a pilot study. Data were summarized into odds ratios, 95% confidence interval (95% CI), and alpha of p<0.05 was considered significant using STATA software (Stata Corp. STATA 12.0, College Station, Texas, USA). The frequency of vitamin D *fok I* gene was determined using percentages. A Chi square test was used to determine the allele and genotype frequency distribution and potential deviation from Hardy Weinberg equilibrium. *Fok I* genotypes were categorized as FF for dominant, Ff heterozygous and ff for homozygous variant.

## Results

### Description of social demographic characteristics of participants

A total of 82 participants were enrolled in the study. Of these, 41 had pulmonary TB while 41 were healthy subjects with no history of the disease. Majority of participants were males (66.0%). Among the participants with TB 73.2% were males while the females were 26.8%. The percentage of HIV/TB patients in this study of 24.4% was lower than the non HIV/TB patients. Thirteen (15.9%) of the 82 participants were HIV seropositive, of these three were from the control group. Most of the TB patients were drivers, farmers, teachers and others. Other lifestyle factors did not show significant variation. Details of the above results are shown in Table 1. Only four (10%) participants were aware of exposure to TB (data not shown).

**Table 1.** The social economic and demographic characteristics of 82 subjects.

Socio-economic characteristics		TB patients, n, (%)	Healthy subjects, n, (%)
**Sex**	M	30 (73.2%)	24 (58.5%)
	F	11 (26.8%)	17 (41.5%)
**Age**	18 – 60 yrs	34.2±12.0	35.2 ±10.7
**Marital status**	Single	18 (43.9%)	19 (46.3%)
	Married	23 (56.1%)	22 (53.7%)
**Shelter**	Large house	19 (46.3%)	25 (60.9%)
	Small house	22 (54.6%)	16 (39.1%)
**Employment**	Employed	34 (83%)	28 (68%)
	Unemployed	7 (7.1%)	13 (31.7%)
**HIV status**	Positive	10 (24.4%)	3 (7.3%)
	Negative	31 (75.6%)	38 (92.6%)
**Daily alcohol**	Yes	5 (12%)	1 (2.5%)
	No	36 (87.8%)	40 (97.5%)
**Smoking**	yes	4 (10%)	0 (0%)
	No	37 (90%)	41 (100%)

### Detection of *fok I* gene PCR amplicons and BLAST identification of *fok I* nucleotides

**Figure 1.**
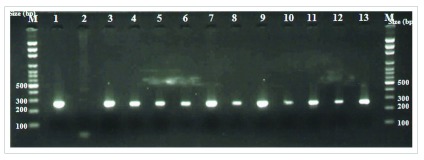
Agarose gel showing PCR amplicons of *Fok I* gene in TB patients and controls. Lanes: M=100bp DNA Ladder, 1=Positive Control, 2=Negative Control, 3–7 and 8–13 = Representative sequencing PCR products from controls and cases respectively.

Following DNA extraction and amplification of the *fok I* gene in all 82 samples the electrophoresis results showed a band between 200 and 300 bps ( Figure 1). The PCR products were then sequenced and a BLAST on the query sequences was done against those in the NCBI Gene bank to confirm the sequences of *fok I.* The gene product was named vitamin D3 receptor isoform VDRB1 vitamin D _3 _receptor isoform VDRA. It showed sequences between 238 bp and 260 bp with a percentage identity between 98–99% (see Dataset 1).

### Frequency distribution of *fok I* genotypes and alleles among TB patients and controls

The frequencies of *Fok I* genotypes, FF, Ff, and ff in TB patients and in the controls were assessed. Table 2 shows the genotype and allele frequency distribution of this study population with their odds ratio, confidence intervals and p values. No significant difference was observed in the distribution of the homozygous genotypes in the study population and the two alleles. FF genotype was used as the reference and other genotypes were compared while the f allele was used as reference to the F allele ( Table 2). The heterozygous Ff genotype was associated with TB, (OR 3.2) since it occurred more in the TB patients than the healthy subjects as shown in Table 2. There was a predominance in the allele distribution of F dominant verse the f recessive allele in both the TB and control group. Both HIV and non HIV individuals predominantly had the FF genotype with only one HIV/TB patient having the recessive ff genotype. Hardy Weinberg equilibrium was tested to establish if the genotype and allele distribution among the TB patients and the control population was in equilibrium. The Chi square test estimated a deviation coefficient of 0.041 in the TB patients and 0.045 in the healthy subjects.

**Table 2.** Genotype and allele distribution of *fok I* gene among TB patients and controls.

*Fok I* Genotypes	TB patients, n = 41	HS, n = 41	OR 95% CI	p value
**FF**	36 (87.8%)	38 (92.6%)	1.0 ref	0.81
**Ff**	3 (7.3%)	1 (2.4%)	3.2 (0.3–31.8)	0.33
**ff**	2 (4.8%)	2 (4.8%)	1.1 ( 0.1–7.8 )	0.96
**Allele**	No of Alelle in TB	No of Allele HS		
**F**	75 (91%)	77 (94%)	1.0 (0.9–1.1)	0.54
**f** **Total No of alleles** **F** **f**	7 (8%) No in TB/Controls 152 (92.7) 12 ( 7.3)	5 (6%)	Ref Ref	0.7

OR =Odds ratio, CI= confidence interval, FF reference genotype and f reference allele

### Genotype *fok I* distribution by gender

An analysis of *fok I* gene was performed to determine the frequency distribution of the *fok I* gene polymorphism among male and female subjects ( Table 3). The frequency distribution of *fok I* FF homozygous dominant genotype was male was 57.3% for male and 32.9 % for females. There was one female with heterozygous Ff genotype while homozygous recessive ff genotype was only found male subjects. These results showed a significant difference in the distribution of the *fok I* genotypes among male and female subjects given a p value of 0.02. When a multivariate analysis was further performed against *fok I*, gender and other variables a p 0.01 was obtained.

**Table 3.** Genotypes of *fok I* based on gender.

*Fok I* Genotypes	Male, n, (%)	Female, n, (%)	P value
**FF** **Ff** **ff**	47 (57.3%) 3 (3.7%) 4 (4.9%)	27 (32.9%) 1 (1.2 %) 0 (0.0%)	0.02

Human VDR fokI gene nucleotide sequences DNA nucleotide sequences of fok I gene showing recognition sites before identification of polymorphisms. Click here for additional data file. Copyright: © 2016 Acen EL et al. 2016 Data associated with the article are available under the terms of the Creative Commons Zero "No rights reserved" data waiver (CC0 1.0 Public domain dedication).

## Discussion

VDR polymorphisms have been studied in different populations and have shown various results. We document for the first time the frequency distribution of VDR polymorphism in the Ugandan population. This study reports a frequency of 87.8% of FF genotype in the Pulmonary Tuberculosis (PTB) patients; however previous studies have reported a 65% frequency of FF genotype among Indian PTB patients, 72% among the Venda of South Africa and 62% among west-Africans. The low frequency of f allele in this population is consistent with another study ^10^ showing that the *f* allele of *Fok I* occurs less frequently in Africans compared to Caucasians and Asians, (Africans 24%, Caucasians 34%, and Asians 51%). The ff genotype frequency of 4.8% was similar to the one described for PTB patients in the African case control study of Gambia Guinea and Guinea Bissau. The ff genotype was reported to be 5% in an Indian healthy population ^11^, while it was 6% in the African American population and 3% in the Venda of South Africa ^12^. The ff genotype was only found among the males in this study. This observation differs from an Indian study showing the FF genotype only in males and a significant difference in the genotypic distribution between males and females. The VDR polymorphism was not in equilibrium with Hardy Weinberg and this could be attributed to the low frequency of the ff genotype. Among other studies that have also reported disequilibrium in the VDR are the studies in the three West African countries ^6^ and a study on the Paraguay population ^8^. Disequilibrium observed in these studies may be due to genotyping errors but was overcome by use of high quality DNA which was achieved by secondary PCR. Other factors are genetic drift and mutations. There was no significant difference in the distribution of *fok I* genotype in different ethnic groups of our study population, (p value 0.30). However a difference was observed when the analysis was based on gender. The low numbers of females in this study may probably be explained by the female sex being a protection against TB according to a study ^13^. Among the 5 % to 10 % of individuals who develop active disease about 70% are males.

## Conclusion

Although the frequency distribution of the homozygous *fok I* genotypes was not significantly different in the Ugandan TB patients and controls the Ff heterozygous genotype appears to be associated with TB in the Ugandan population. Therefore further studies are needed to elucidate this relationship.

## Data availability

The data referenced by this article are under copyright with the following copyright statement: Copyright: © 2016 Acen EL et al.

Data associated with the article are available under the terms of the Creative Commons Zero "No rights reserved" data waiver (CC0 1.0 Public domain dedication). 

F1000Research: Dataset 1. Human VDR fok I gene nucleotide sequences, 10.5256/f1000research.9109.d130420 ^14^

## Consent

Written informed consent to participate in the study and publish these data was obtained from all participants.

## Ethical considerations

Permission to carry out the study was obtained from the Research and Ethics Committee Mulago Hospital, (ref MREC: 329), the Institutional Review Board of the School of Biomedical Sciences Higher Degrees Research and Ethics committee, (ref SBS 108), and from the Uganda National Council of Science and Technology (ref No.1431). Before investigations, informed consent was obtained from the study subjects. Patients’ personal information was kept confidential by using serial codes instead of names on the questionnaire. 
