# Supplementary material for: The frequency distribution of vitamin D Receptor fok I gene polymorphism among Ugandan pulmonary TB patients
Source: F1000Res. 2016 Jul 29;5:ISCB Comm J-1890. [Version 1] doi: 10.12688/f1000research.9109.1 (PMC5022702; doi:10.12688/f1000research.9109.1)
Supplement: Peer review history

# Peer review: Referee response for version 1

**DOI:** 10.5256/f1000research.9803.r16253

**Published:** 2016-09-13

**Authors:** Range Nyagosya

**Affiliations:** 1 Muhimbili Research Centre, National Institute For Medical Research (NIMR), Dar es Salaam, Tanzania

The vitamin D receptor plays an important role in the immune system and the roles have widely been studied. Several studies have been conducted to assess the frequency distribution as well as roles of Vitamin D’s in various diseases conditions; including; multiple sclerosis, cancer, diabetes and TB. However, the findings have been varying and inconclusive, imposing the need of carrying further investigations in different populations and settings to determine the roles and frequency distrition. It was on this varying state of Vitamin D that the authors, Ester *et al*, conducted a study in Uganda aiming to investigate the frequency distribution of the VDR gene ( *fok *I) polymorphisms in pulmonary TB patients compared to controls in Ugandan population.

 The article though short and with relatively small sample size of 82 (TB patients and health controls) is well written, appropriate description is given covering all sections; introduction, objectives methods, results, discussions and conclusion. The study found no significance difference in the frequency distribution of homozygous –dominant (FF) and homozygous receive (ff) genotype among TB patients and controls. However, heterozygous (Ff) genotype was more frequent among TB patients compared to controls (7.3% vs 2.4%).  The author concluded that Ff gene may be associated with TB because it occurred more in TB patients than in health controls.

**Specific comment:**

 In the discussion section the author gave references to other studies done elsewhere on the frequency of FF and ff genotypes, however, references on the frequency of heterozygous (Ff) is missing and should be added in the discussion section. In this study the difference was observed on this gene for the Ugandan studied population hence reference to other studies findings is important.

I have read this submission. I believe that I have an appropriate level of expertise to confirm that it is of an acceptable scientific standard.

# Peer review: Referee response for version 1

**DOI:** 10.5256/f1000research.9803.r15526

**Published:** 2016-08-31

**Authors:** Lori B. Chibnik

**Affiliations:** 1 Department of Epidemiology, Harvard T.H. Chan School of Public Health, Boston, MA, USA; 2 Department of Medicine, Harvard Medical School, Boston, MA, USA

This manuscript describes a case-control study carried out at the Mulago National Referral and Teaching Hospital to assess the possible association between the *fok *I gene and TB diagnosis.  The cases were defined as newly diagnosed cases of TB and were matched to TB negative controls taken from health workers at the hospital.  The minor allele frequency (MAF) was smaller than expected with the overall MAF = 7%. While some differences were found between TB cases and controls, none were statistically significant.  It is a fairly straight forward analysis and Dr. Acen and colleagues made a valiant effort to explore this question.

 Some comments:

1. The controls were chosen from health workers and trainees at the hospital (age and sex matched).  This brings in some confounding as health workers and medial trainees tend to be different than the general population in terms of education, SES, etc. some of which include risk factors for TB.  This needs to be addressed in the discussion as a limitation.   Also, were the controls tested to confirm they do not have TB, this should also be mentioned.
   
  1. I am assuming the health workers are the controls, the wording is a bit strange.  The manuscript needs to be proof-read by a native or more fluent English speaker
2. The authors state that cases and controls were matched on sex, but the numbers (table 1) do not show this.  There are 30 males in the TB group and only 24 in the controls. 
3. The main result of this paper is the OR of 3.2 presented in table 2.  The authors claim that this is a real effect and shows a possible relationship between TB and the *fok I* gene.  They neglect to mention the 95% CI that includes the null (0.3 – 31.8) in text which is misleading.  With such small numbers and a non-significant effect this is not a meaningful finding.  It argues that more research is needed, but it is hard to argue that there is a real effect shown in these data.
4. Statistical Analysis section and Table 2:
   
  1. The authors list numbers (78.3 and 83.5) as means for use in a power calculation.  What do these numbers stand for?  If they are allele frequencies then that should be stated.
  2. With such low frequencies in the some of the cells, a chi-square test is not appropriate. A Fisher’s exact test should be used to determine p-values
  3. Why is FF used as a reference genotype but f used as reference allele?  This makes the interpretation a bit more confusing. 
  4. How were the p-values calculated for Table 2?  This needs to be indicated.  What is the p-value for the reference groups from? 
5. Table 3
   
  1. The analyses based on sex needs to be described in the statistical analysis section.
  2. What was the impetus to compare frequencies based on sex?  It seemed to come out of nowhere.
6. The authors should focus more on the differences in MAF for *fok I* gene in their population compared to other published populations of different ancestry. 

I have read this submission. I believe that I have an appropriate level of expertise to confirm that it is of an acceptable scientific standard, however I have significant reservations, as outlined above.

# Peer review: Referee response for version 1

**DOI:** 10.5256/f1000research.9803.r15525

**Published:** 2016-08-19

**Authors:** Simon H. Martin

**Affiliations:** 1 Department of Zoology, University of Cambridge , Cambridge, UK

Acen *et al*. investigated whether the fok I polymorphism in the vitamin D receptor gene is associated with tuberculosis in 82 (41 infected and 41 uninfected) Ugandans.

 This was a pilot study with a fairly small sample size, and I have evaluated it as such. In general, I see no fundamental flaws in the study design. However, I have some serious concerns about the statistical analyses and interpretation thereof, as well as several minor concerns, as detailed below.

 Major concerns

1. Association of heterozygous Ff with TB
  In the Results it is claimed that "The heterozygous Ff genotype was associated with TB, (OR 3.2) since it occurred more in the TB patients than the healthy subjects. The Abstract also states that "The Ff heterozygous genotype distribution appeared more in TB patients than in controls". And the discussion says "the Ff heterozygous genotype appears to be associated with TB in the Ugandan population". I think these claims are misleading, as the result was not statistically significant at all. In fact, when you consider the raw numbers (3 of the 41 TB patients were Ff and 1 of the 41 healthy samples were Ff), I think it is clear that there is no evidence in this data for an association between Ff and TB status. I feel that the text should be revised to acknowledge the lack of statistical support for this claim.
2. Association between genotype and gender
  The section "Genotype fok I distribution by gender" and Table 3 is not very clear. It appears to state that genotype FF occurred at a frequency of 57.3% in males and 32.9% in females, but this seems to be a mistake. Table 3 shows that FF occurred in 47 of 54 males (87%) and 27 of 28 females (96.4%). There is no information about the statistical test performed, but the genotype frequencies look to me to be too similar to justify the p-value of 0.02 in Table 3. There is also no information given about the multivariate analysis mentioned. Therefore, I recommend that this entire section needs to be revised, with clearer descriptions of the statistical analyses and correction of the genotype frequencies.
3. Deviation from Hardy-Weinberg Equilibrium
  More details of this chi-square test are required. As far as I can tell, because the frequency of the f allele is so low, the expected number of ff samples according to HWE is zero, so I'm not sure how the chi squared test could be performed. There are methods to test for a deviation from HWE that don't have the same problem with rare alleles. For example, see Wigginton *et al. *(2005).

Minor concerns

1. Abstract line 1 – Mycobacterium tuberculosis is not TB, it is the causative agent of TB.
2. Introduction Par 1 - “Similarly the widely studied and functional single nucleotide polymorphism (SNP) rs2228570 of VDR fok I gene has shown variations.” The meaning of this sentence is not clear.
3. Dataset 1 only includes 35 sequences. Where are the others?
4. References missing in a few places:
  - Introduction Par 1 - “Vitamin D insufficiency is common worldwide and recent studies have shown that vitamin D insufficiency is associated with a higher risk of active TB”
  - Discussion Par 1 - “The ff genotype frequency of 4.8% was similar to the one described for PTB patients in the African case control study of Gambia Guinea and Guinea Bissau.”

I have read this submission. I believe that I have an appropriate level of expertise to confirm that it is of an acceptable scientific standard, however I have significant reservations, as outlined above.

## References

[1] WiggintonJECutlerDJAbecasisGR: A note on exact tests of Hardy-Weinberg equilibrium. *Am J Hum Genet*.2005;76(5) : 10.1086/429864 887-93 10.1086/429864 15789306PMC1199378
